# Free Radical Scavenging Activity: Antiproliferative and Proteomics Analyses of the Differential Expression of Apoptotic Proteins in MCF-7 Cells Treated with Acetone Leaf Extract of *Diospyros lycioides* (Ebenaceae)

**DOI:** 10.1155/2015/534808

**Published:** 2015-09-17

**Authors:** M. C. Pilane, V. P. Bagla, M. P. Mokgotho, V. Mbazima, T. M. Matsebatlela, I. Ncube, L. Mampuru

**Affiliations:** Department of Biochemistry, Microbiology and Biotechnology, Faculty of Science and Agriculture, University of Limpopo, Turfloop Campus, Private Bag X1106, Sovenga, Limpopo 0727, South Africa

## Abstract

Breast cancer is the most common cancer in South Africa. The acetone leaf extract of *Diospyros lycioides* was evaluated qualitatively and quantitatively for its antioxidant potential using DPPH assay and nitric oxide radical scavenging effect, while the viability of MCF-7 cells was evaluated using the MTT. MCF-7 treated cells were stained with Hoechst 335258 dye and annexin-V-FITC to be evaluated for apoptotic effect of the extract, while mRNA expression levels of apoptotic genes were assessed by quantitative real-time PCR and deferential protein expression levels using 2D gel electrophoresis and mass spectrometry. Results revealed presence of antioxidant constituents in the extract. Extract was shown to be cytotoxic in a concentration- and time-dependent manner. Cytotoxicity was demonstrated to be due to apoptosis, with 70% of the extract-treated cells being annexin-V-positive/PI negative at 48 hours. The extract was also shown to upregulate the expression of p53 gene with concomitant downregulation of the Bcl-2 antiapoptotic gene while differentially expressed proteins were identified as enolase, pyruvate kinase, and glyceraldehyde-3-phosphate. The extract in this study was shown to induce apoptosis at an early stage which makes it an ideal source that can be explored for compounds that may be used in the treatment and management of cancer.

## 1. Introduction

Breast cancer remains a considerable health burden. It affects more than 1.3 million women worldwide annually and is responsible for about 14% of cancer-related deaths [1]. Indeed, the incidences of breast cancer have increased over the past decades and a substantial rise is projected in the coming years [2]. Breast cancer is a heterogeneous and polygenic disease which is influenced by epigenetic mechanisms that affect the transcriptomes, proteomes, and metabolomes.

The metabolism of cancer cells has been extensively investigated using numerous approaches leading to the unveiling of diverse mechanisms that are involved in metabolic reprogramming of cancer cells. Cells undergoing apoptosis show distinct characteristic features which include cell shrinkage and rounding, due to the breakdown of the proteinaceous cytoskeleton by caspases, cytoplasmic condensation, chromatin aggregation, and partitioning of the nucleus and cytoplasm into membrane-bound organelles known as apoptotic bodies [3]. Apoptosis can be induced by a variety of physiological conditions as well as damage or therapy-associated agents. Two major pathways of apoptosis have been identified, namely, the death receptor (extrinsic) and the mitochondrial (intrinsic) pathways.

The intrinsic pathway can be triggered by a number of stimuli such as heat shock, UV radiation, and DNA damage. In this pathway, members of the Bcl-2 family of proteins affect the mitochondrial function and regulate the release of apoptosis-activating factors. Antiapoptotic members of Bcl-2 family such as Bcl-2 and Bcl-xL act to preserve mitochondrial integrity by suppressing the release of cytochrome C [4]. In contrast, proapoptotic members, Bax, Bid, and Bad, induce the release of cytochrome C resulting in mitochondrial dysfunction. Bax, which normally resides in the cytosol, translocates to the mitochondria when triggered by certain stimuli. Translocation of Bax is then followed by the release of cytochrome C and subsequent caspase activation [4].

*p*53 on the other hand is a tumour suppressor gene and is one of the most commonly mutated genes involved in many types of cancers. It is involved in cell-cycle control, proliferation, and apoptosis. Its main role is to maintain the integrity of the genome due to its ability to regulate various aspects of cell growth and apoptosis.* p53* can arrest damaged cells at the G1 phase of the cell division cycle, thus providing time for DNA to be repaired or induce apoptosis if the damage is too extensive [5]. The wild-type* p*53 induces apoptosis in injured or damaged cells and prevents the proliferation of transformed cells [6]. As such, mutation in the* p53 *gene can result in the suppression of apoptotic cell death leading to carcinogenesis.

*Diospyros lycioides* (DL) (Ebenaceae), commonly known as the bluebush, is distributed in central Africa, Southern Tanzania, and southern parts of Africa, including South Africa. It is a small tree that grows up to 7 metres tall and grows well in tropical areas. The leaves are an important browse for both domestic and wild animals while the fruits are fermented to distil alcohol. Its crushed roots have been documented to be used traditionally in the treatment of colds, coughs, and infertility in women [7]. A research paper documented by Cai et al. [8] reports the methanol extract of this plant to inhibit the growth of selected oral pathogens. Preliminary and unpublished studies from our laboratory have also shown the acetone leaf extract of this plant to possess constituents with antioxidant activity and antiproliferative effect on the growth of HL-60 promyelocytic leukaemia cell line, a finding that provoked renewed interest in investigating the anticancer potential of this plant and the elucidation of the mechanism(s) responsible for the observed effects in MCF-7 breast cancer cells.

All cancer cells acquire the ability to grow and divide in the absence of appropriate signals or in the presence of inhibitory signals. Since no published report to our knowledge is available on the antiproliferative and apoptotic effects of DL on MCF-7 cells, this study was aimed at determining the* in vitro* anticancer mechanism(s) of the acetone extract of this plant on the selected cell line.

## 2. Materials and Methods

### 2.1. Plant Collection and Extraction

The leaves of DL were collected during summer from Bolahlakgomo area (Lepelle-Nkumpi Municipality, Limpopo Province, South Africa) in February and voucher specimen (UNIN 111076) is deposited at the Larry Leach Herbarium of the University of Limpopo. Collected leaves were dried at room temperature for two weeks and ground into a fine powder using a blender. The ground plant material was exhaustively extracted using acetone (1 g/10 mL) on a shaker at 200 rpm. The extracted plant material was filtered using a 0.22 *μ*m pore size filter paper (Whatman Inc.) into preweighed glass beakers. The samples were concentrated at 40°C using a Buchi rotor vapour R-205 (Labotec) and dried under a stream of cold air at room temperature.

### 2.2. Thin Layer Chromatography (TLC) Profiling

Extracted plant material (10 *μ*L) dissolved in acetone (10 mg/mL) was spotted onto TLC plates (Macherey-Nagel, Düren, Germany) and eluted in different solvent systems, namely, EMW (ethyl acetate : methanol : water, 10 : 1.35 : 1 v/v/v), BEA (benzene : ethanol : ammonia, 18 : 2 : 0.2, v/v/v), and CEF (chloroform : ethyl acetate : formic acid, 10 : 8 : 2 v/v/v). The plates were sprayed with vanillin-sulphuric acid reagent (0.1 g vanillin, 28 mL methanol, and 1 mL sulphuric acid) and developed in an oven at 110°C for 5 min [10].

### 2.3. Qualitative Antioxidant Activity

The extract was further screened for the presence of antioxidant constituents on TLC plates following elution in the solvent systems (Section 2.2) and sprayed with 0.2% of 2, 2-diphenyl-1-picrylhydrazyl (DPPH) in methanol for visualization of antioxidant compounds present in the extract [11].

### 2.4. DPPH Radical Scavenging Activity Assay

Quantitative antioxidant activity of the acetone leaf extract of DL was evaluated by adding 100 *μ*L of the extract (1000 *μ*g/mL) dissolved in DMSO into 96-well plate and twofold serial diluted to obtain concentrations ranging between 31.25 and 1000 *μ*g/mL [12]. Equal volumes of 0.2% DPPH were added to the plate and incubated at room temperature for 30 minutes. After incubation, the absorbance was read at 490 nm using a microtiter-plate reader (Model 550, Bio-Rad Laboratories, California, USA). Vitamin C was used as a positive control and DMSO as a blank. The scavenging activity of the extracts was calculated as follows:

(1) Percentage  inhibition=A490  of  blank−A490  of  sampleA490  of  blank×100.

### 2.5. Nitric Oxide (NO) Radical Scavenging Effect

In the presence of oxygen, sodium nitroprusside produces free radicals such as nitric oxide and nitrates which are then measured by Griess reagent [13]. Various concentrations (25 to 500 *μ*g/mL) of the extract were prepared and incubated for 3 h. An equal volume of Griess reagent (1% sulfanilamide and 0.1% ethylene diamine dihydrochloride in 5% H_3_PO_4_) was added and the absorbance read at 550 nm using a microtiter-plate reader (Model 550, Bio-Rad Laboratories, California, USA).

### 2.6. MTT Assay

The MTT assay was used to determine the cytotoxic effects of the extract on MCF-7 cell lines. This assay was used to assess cell viability based on the reduction of MTT by mitochondrial succinate dehydrogenase enzyme of the viable cells to a purple formazan product [14]. The cells were suspended in growth medium (DMEM supplemented with 10% FBS and 1% penicillin-streptomycin-neomycin cocktail, PSN) and seeded in 96-well plates at a density of 2 × 10^5^ cells/well. The cells were incubated at 37°C for 24 h to attain 80% confluence. Stock concentration of extract (10 mg/mL) dissolved in 1% DMSO and made to a final concentration ranging from 25 to 500 *μ*g/mL was prepared. Cells were exposed to the varying concentrations while curcumin-treated cell (20 *μ*M) served as a positive control. The plates were incubated at 37°C for 24, 48, and 72 h. After incubation, a volume of 50 *μ*L of MTT (5 mg/mL) was added to each well and incubated at 37°C for 3 h. The media was aspirated and cells were washed with phosphate-buffered saline (PBS). The cells were dissolved in 100 *μ*L DMSO and absorbance was measured at 595 nm using a microtiter-plate reader (Model 550, Bio-Rad Laboratories, California, USA). The percentage of viable cells was calculated as follows:

(2) Percentage  viability=A595  of  control−A595  of  sampleA595  of  control×100.

Cytotoxic concentration of the extract was extrapolated from a linear regression graph.

### 2.7. Nuclear Morphological Changes Associated with Apoptosis

The nuclear morphological changes of apoptotic cells were assessed by staining the cells with DNA-binding dye, Hoechst 335258. The cells were seeded at a density of 2 × 10^5^ cells/well and allowed to attach overnight on a microscope slide. The cells were then exposed to various cytotoxic concentrations of the extract for 48 h. Curcumin (20 *μ*M) was used as a positive control. After treatment the cells were washed once with cold PBS and incubated with 20 *μ*g/mL of Hoechst 33258 dye for 30 min in the dark. The solution (dye) was discarded and the cells were washed with PBS, viewed, and photographed under a fluorescence microscope (Nikon Eclipse Ti H600L, Japan).

### 2.8. Annexin V Apoptosis Detection Assay

The detection and measurement of the percentage of apoptotic cells was analysed by annexin V-FITC staining and flow cytometry. After treatment with cytotoxic concentration of extract for 24 and 48 h, cells were harvested by centrifugation (1000 rpm) and fixed with 70% ethanol for 30 min at 4°C. Annexin V-FITC/propidium iodide stain was added to the cells and incubated for 30 min. After incubation, cells were washed with PBS and centrifuged at 1000 rpm. The pellet was suspended in PBS and the stained cells were analysed using a flow cytometer (BD FACSCalibur System, Becton Dickinson (Pty) Ltd., CA, USA).

### 2.9. Quantitative Real-Time Polymerase Chain Reaction (qrt-PCR)

The mRNA expression levels of apoptotic genes were assessed by using quantitative real-time PCR. Cells were treated with various concentrations of the extract and then harvested at 24 and 48 h time intervals. The cells were then washed twice with ice-cold PBS and total RNA was isolated using a high pure RNA isolation kit (Roche Diagnostics GmBH, Mannheim, Germany) according to the manufacturer's protocol. The purity of RNA was determined by the ratio of *A*_260_/*A*_280_. The total cellular RNA was synthesised into cDNA using the High Capacity cDNA Reverse Transcription (Applied Biosystems, CA, USA). Briefly, 1 *μ*g of RNA was added to a reverse transcription mixture (1x PCR buffer, reverse transcriptase (2.5 units), 0.5 mM deoxynucleotide triphosphates (dNTPs), and random RT-PCR primers) and incubated for 10 min at room temperature. After incubation, the mixture was placed in a thermal cycler, Rotor-Gene 6000 system (Corbett Research, Australia), and further incubated at 42°C for 15 min and then heated to 95°C for 5 min and cooled at 4°C for 5 min. The cDNA was quantified by measuring the absorbance at 260 nm. For quantitative real-time PCR, cDNA was amplified in 25 *μ*L reaction mixture (1x SensiMix master mix, 1x Sybr green, and 0.8 *μ*M of forward and reverse primers). The cycling conditions for polymerase reaction were set at 95°C, 30°C (for glyceraldehyde-phosphate dehydrogenase (*GAPDH*)), and 40°C (for* bax*,* bcl-2, p21*, and* p53*) for 10 min and annealing temperatures at 58°C (for* bcl-2, p21*, and* p53*) and 60°C (for* bax*) for 15 sec, elongation at 72°C for 20 sec, and denaturation at 95°C for 10 sec. The molecular concentration of the template was quantified using the standard curve method for relative quantification.  Table 1 represents the primer sequences used in the real-time PCR assay.

### 2.10. Protein Isolation

The experimental culture samples were collected and harvested by centrifugation at 1000 rpm and then washed with PBS to remove any residual media components. After washing, the cells were lysed using lysis buffer (10 mM Tris, pH 6.8, 1% SDS, 1% Nonidet P-40, 100 mM sodium chloride, dH_2_O, and 1 M DTT). The tubes were vortexed to disrupt cell membrane and the supernatant was collected by centrifugation at 12 500 ×g for 20 min at 4°C. Proteins were quantified using BCA protein assay (Thermo Scientific, Rockford, USA) according to the manufacturer's protocol.

### 2.11. Two-Dimensional (2D) Gel Electrophoresis

Volumes of 140 *μ*L of protein samples were diluted in sample rehydration buffer (8 M Urea, 20–100 mM of DTT and ampholyte solution) and each strip was rehydrated for the standard rehydration time of 1 h. The IPG strips containing samples with different protein concentrations were loaded in the IPG runner. The sample loading wells were sealed with a sealing tape and incubated for 1 h overnight at room temperature. The isoelectric focusing of the strips was performed in a three-step manner: the initial voltage was set at 200 V for 20 min, then increased to 450 V for 15 min followed by 750 V for another 15 min, and lastly set at 2000 V for 90 min. The current limit was at 50 *μ*A per strip. After focusing, the strips were equilibrated in buffer (1x LDS sample buffer and sample reducing agent) for 15 min and then alkylated in a solution containing 1x LDS sample buffer and iodoacetamide for 15 min.

The proteins were separated by 15% sodium dodecyl sulphate-polyacrylamide gel electrophoresis (SDS-PAGE) run at 150 V for 20 min and 200 V for 30 min. The technique was performed following the manufacturer's protocol (Zoom IPGRunner System, Invitrogen, UK). The gels were then stained with coomassie brilliant blue R-250 for visualisation of spots. The gel images were analysed using Quantity One Software and PDQuest version 8.0.1 2D gel analysis software and the protein spots were detected following the manufacturer's protocol. The sequences of the identified spots were subjected to Basic Local Alignment Search Tool (BLAST).

### 2.12. Statistical Analysis

The experimental values are expressed as ± standard deviation (SD). The statistical significance (*p* ≤ 0.05) was calculated by one-way ANOVA and Dunnett tests using GraphPad InStat software.

## 3. Results

### 3.1. TLC Profile and Qualitative Antioxidant Activity

**Figure 1:**
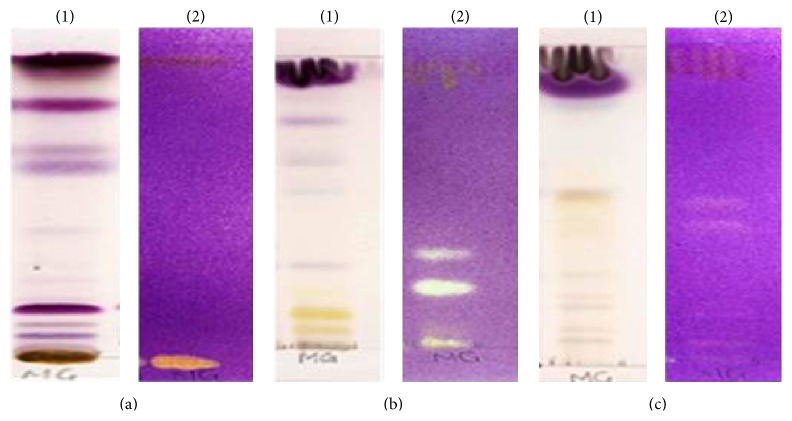
TLC finger print profile and presence of antioxidant constituents in the acetone crude extract of DL, developed in (a) (BEA (18 : 2 : 0.2 v/v/v)), (b) (CEF (10 : 8 : 2 v/v/v)), and (c) (EMW (10 : 1.35 : 1 v/v/v)) solvent systems. The plates were sprayed with vanillin-sulphuric acid reagent (1) and 0.2% DPPH (2) for visualization of compounds with antioxidant activity.

Thin layer chromatographs were developed in different solvent systems BEA (18 : 2 : 0.2 v/v/v), CEF (10 : 8 : 2 v/v/v), and EMW (10 : 1.35 : 1 v/v/v) and sprayed with vanillin. Good separation of constituents eluted in CEF revealed the presence of constituents with a range of colours suggestive of the presence of different compounds (Figure 1(1)) such as steroids, terpenoids, and flavonoids. Figure 1(2) shows plates that were sprayed with DPPH with antioxidant compound evident against a purple background. The antioxidant constituents were shown to be very polar and were best separated in the intermediate polar mobile eluent system.

### 3.2. Free Radical Scavenging Activity Assays

**Figure 2:**
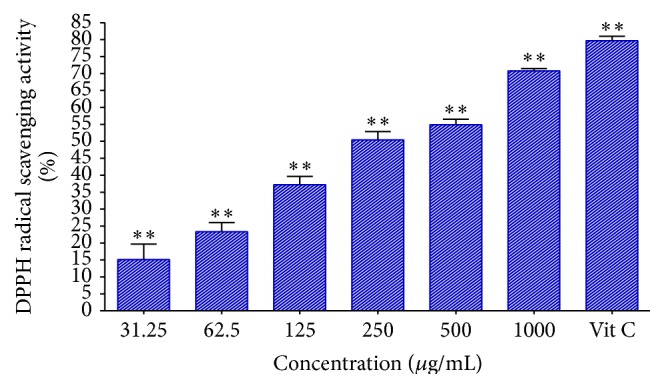
The DPPH radical scavenging activity of acetone crude extract of DL. Vitamin C was used as a positive control.  ^*∗∗*^Statistically significant (*p* < 0.01).

**Figure 3:**
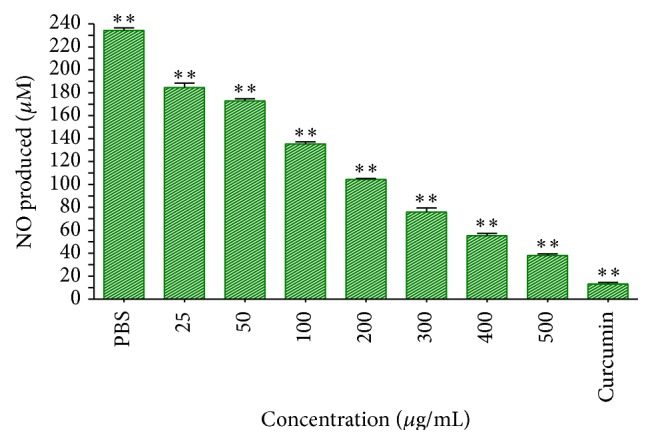
The nitric oxide free radical scavenging effect of the crude extract of DL. Curcumin (20 *μ*M = 7.36 *μ*g/mL) was used as a positive control.  ^*∗∗*^Statistically significant (*p* < 0.01).

The free radical scavenging activities of the extract were assayed using quantitative DPPH and nitric oxide scavenging activity assays. It appears that the acetone leaf extract of DL possesses constituents with hydrogen donating abilities, indicative of their antioxidant activity in a concentration-dependent manner (Figure 2). An increase in concentration is shown to increase the scavenging activity of the extract in the DPPH radical scavenging assay whereas the reverse was the case in the nitric oxide free radical scavenging assay as measured by Griess reagent (Figure 3). High accumulation of nitric oxide in tissues causes pathologies such as hypertension, cardiovascular dysfunctions, neurodegenerative diseases, arthritis, asthma, and septic shock in human.

### 3.3. Antiproliferative Effects of the Acetone Extract of DL on MCF-7 Cell Lines

**Figure 4:**
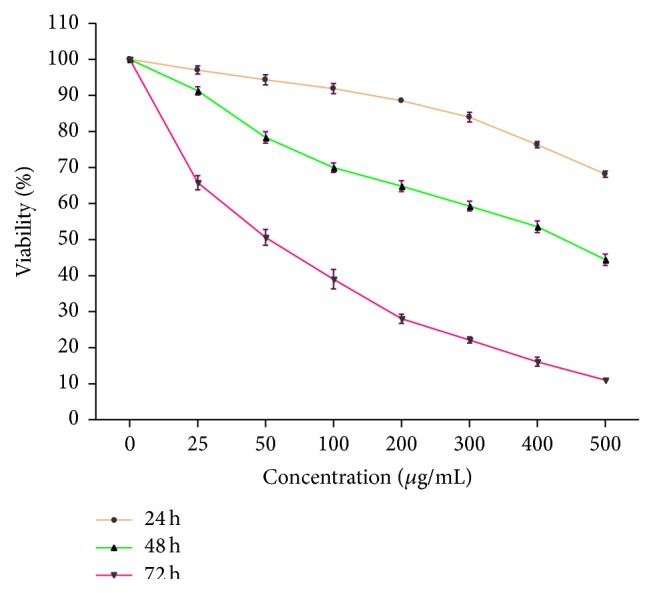
The effect of acetone leaf extract of DL on the viability of MCF-7 cells treated with various concentrations of the extract. The results represent the mean of three independent experiments, each done in duplicate.

The antiproliferative effect of the acetone extract on MCF-7 cell line was determined by MTT assay. This assay is based on the capacity of the mitochondrial enzyme, succinate dehydrogenase, in viable cells to transform MTT tetrazolium salt into a blue formazan product. Inhibition of the proliferation of MCF-7 cells by the extract was shown to be concentration-dependent at the various time intervals tested. At prolonged exposure time of 72 h, the extracts at 50 *μ*g/mL were shown to inhibit the viability of cells by about 50%. Cells exposed for 48 h at the highest concentration of 500 *μ*g/mL were shown to inhibit their viability at about 50% (Figure 4). The IC_50_ of the extract was determined to be 65 *μ*g/mL following 48 h of exposure.

### 3.4. Induction of Apoptosis by DL Extract in MCF-7 Cells

**Figure 5:**
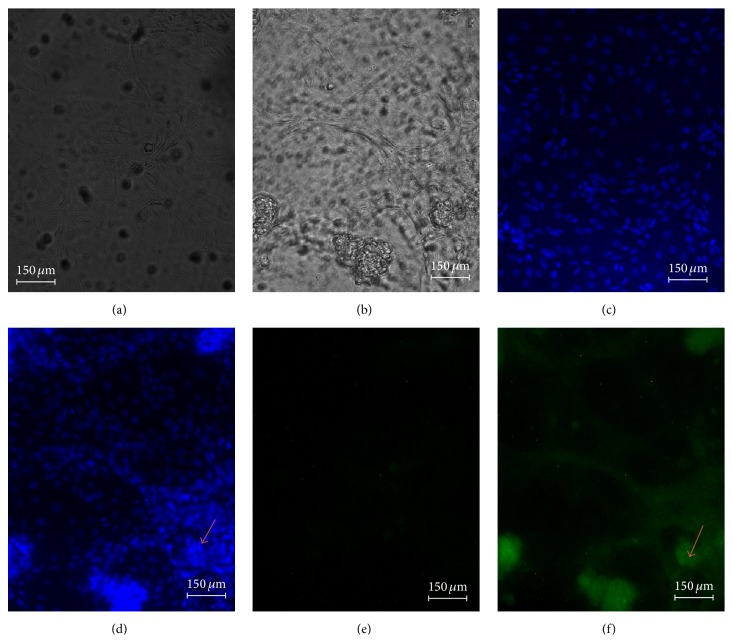
The nuclear morphological changes of MCF-7 cells treated with 65 *μ*g/mL of the DL extract, (a) = untreated cells, (b) = cell + 65 *μ*g/mL extract treatment, (c) = untreated cells + Hoechst 33258, (d) = 65 *μ*g/mL treatment + Hoechst 33258, (e) = untreated cells + annexin V-FITC, and (f) = 65 *μ*g/mL treatment + annexin V-FITC, for 48 h viewed under fluorescence microscope.

To determine whether the antiproliferative activity and the decrease in cell viability of MCF-7 cells are a result of induction of apoptosis, the nuclear morphology changes in characteristic of apoptosis were assessed using the DNA-binding dye (Hoeschst 33258) and annexin V-FITC. A representative micrograph is presented in Figure 5. The morphological changes of cells were evident by the presence of condensed chromatin and fragmented nuclei as shown by the light-blue fluorescence in treated cells and green fluorescence due to the externalisation or exposure of phosphatidylserine, which embodies the early phase of apoptosis.

### 3.5. Flow Cytometry

**Figure 6:**
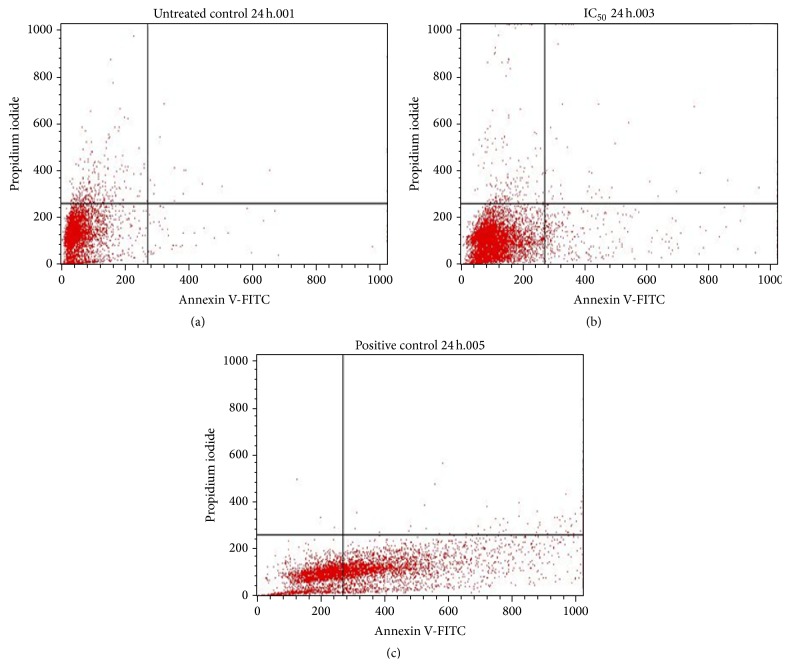
Flow cytometric analysis of MCF-7 cells for apoptosis. Cells were stained with annexin V-FITC and propidium iodide for 24 h, where (a) = untreated cells, (b) = IC_50_ (65 *μ*g/mL), and (c) = positive control (curcumin).

**Figure 7:**
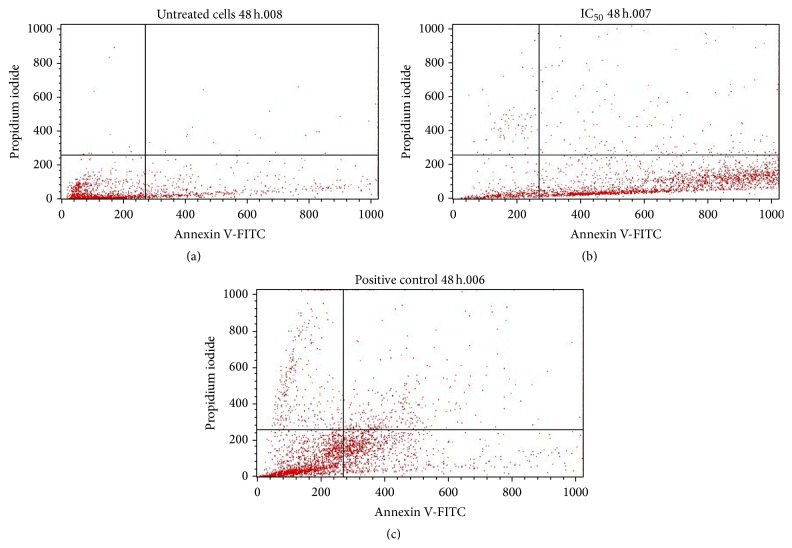
Flow cytometric analysis of MCF-7 cells for apoptosis. Cells were stained with annexin V-FITC and propidium iodide for 48 h, where (a) = untreated cells, (b) = IC_50_ (65 *μ*g/mL), and (c) = positive control (curcumin).

**Figure 8:**
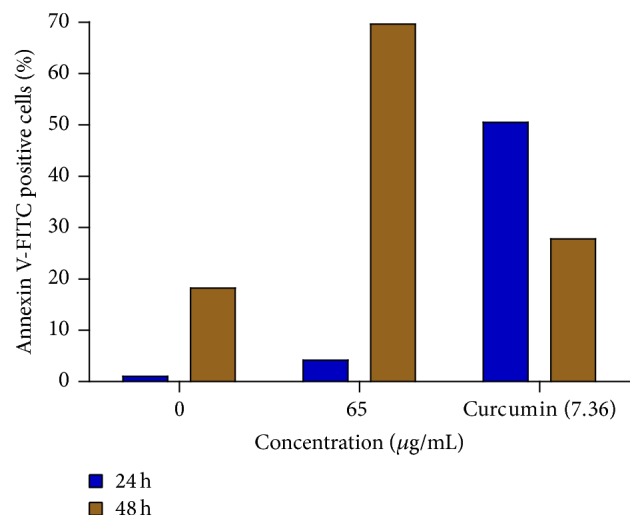
Percentage of apoptotic MCF-7 cells after treatment with 65 *μ*g/mL of the extract for 24 and 48 h. The 0 *μ*g/mL represents untreated cells, while curcumin (7.36 *μ*g/mL) represents the positive control.

To quantitatively ascertain the percentage of cells within the population that were actively undergoing apoptosis, untreated and treated MCF-7 cells were stained using annexin V-FITC/propidium iodide (PI) and evaluated by flow cytometry. As shown in Figures 6(a) and 7(a), only a small percentage of untreated cells were annexin V-FITC positive after 24 and 48 h of incubation. In contrast, the percentage of annexin V-FITC positive cells increased after treatment with 65 *μ*g/mL of the extract after 24 h (Figure 6(b)) and 48 h (Figure 7(b)) of incubation. The results demonstrated that the extract induced apoptosis of MCF-7 cells in a time-dependent manner. A graphical representation of the percentage of annexin V-FITC positive cells after treatment with 65 *μ*g/mL of extract for 24 and 48h is presented in Figure 8.

### 3.6. The Effects of DL Extract on* p53*,* bcl*-2, and* bax* mRNA Expression

**Figure 9:**
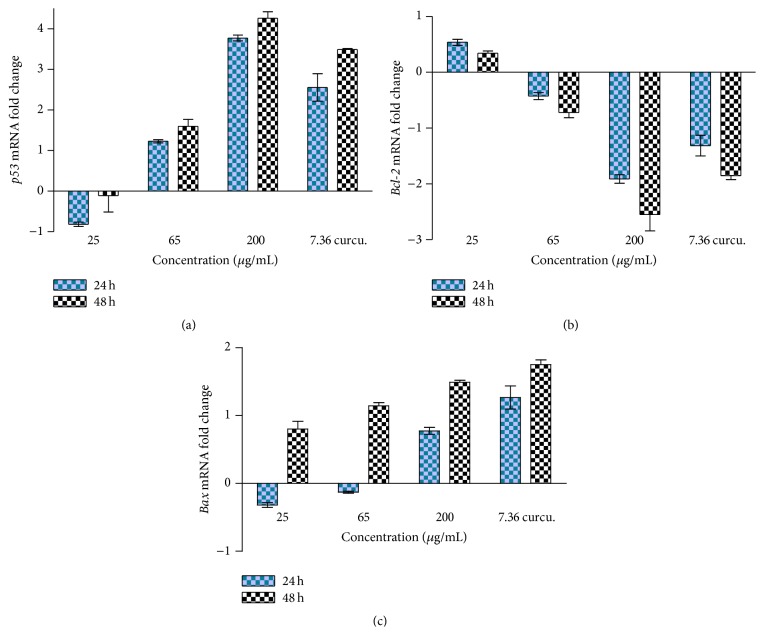
The effects of different concentrations of DL extract on the mRNA expression levels of* p*53 (a),* bcl*-2 (b), and* bax* (c) in MCF-7 cells as determined by real-time PCR.

The effect of DL extract on mRNA expression levels of the apoptotic genes,* p*53,* bcl*-2, and* bax*, was assessed by quantitative real-time PCR. It was observed that an increase in the concentration of the extract was directly proportional to an increase in* p*53 expression levels with time of exposure (Figure 9(a)). On the contrary, exposure of MCF-7 cells to the extract resulted in the downregulation of* bcl*-2 mRNA levels in a time- and dose-dependent manner (Figure 9(b)), while the* bax *mRNA expression level was shown to be upregulated (Figure 9(c)).

### 3.7. Two-Dimensional Gel Electrophoresis

**Figure 10:**
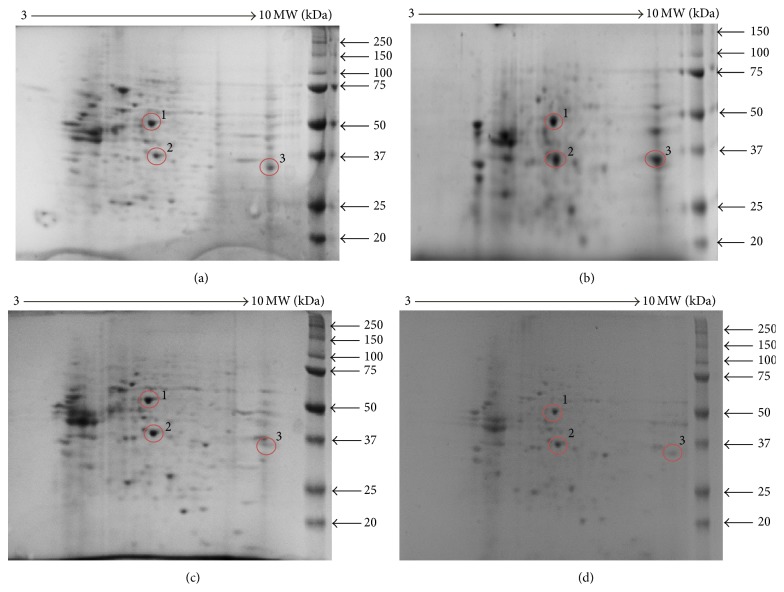
2D-PAGE images of proteins extracted from MCF-7 cells (a) untreated and (b) 25 *μ*g/mL of DL extract. Proteins were separated in the first dimension (1D) at pH 3–10 and on a 15% SDS-PAGE in the second dimension. Protein spots (circled) were further analysed by MALDI-TOF mass spectrometry. 2D-PAGE images of proteins extracted from MCF-7 cells (c) 65 *μ*g/mL of DL extract and (d) positive control, curcumin. Proteins were separated in the first dimension (1D) at pH 3–10 and on a 15% SDS-PAGE in the second dimension. Protein spots (circled) were further analysed by MALDI-TOF mass spectrometry. The peptide sequences of the identified spots are presented in Spot 1, Spot 2, and Spot 3.

The differentially expressed protein levels of MCF-7 cells exposed to 25 *μ*g/mL and 65 *μ*g/mL that can be illustrated by the regulated intensity of spots after staining were assessed by 2D gel electrophoresis. The highly intense bands show the high expression of the protein and vice versa.  Figure 10 shows the expression of proteins isolated from the treatment of the cells with various concentrations of the extract. Figures 10(a) and 10(b) represent 2D-PAGE images of proteins extracted from MCF-7 cells following treatment with 25 *μ*g/mL of DL extract. Proteins were separated in the first dimension (1D) at pH 3–10 and on a 15% SDS-PAGE in the second dimension and analysed by MALDI-TOF mass spectrometry. Figures 10(c) and 10(d) show the low expression levels of Spot 3 as opposed to its high intensity in Figures 10(a) and 10(b), suggestive of inhibition of the expression level of Spot 3 at the concentration of 65 *μ*g/mL.

The spots on gels were analysed by MALDI-TOF mass spectrometry and the highlighted spots were subjected to tryptic digestion and subsequent peptide sequencing. The highlighted sequences (Table 2) indicate the conserved regions of the peptide sequences. The sequences matched three proteins: enolase-3 (beta-muscle) isoform CRA_b (*Homo sapiens*) (Spot 1), pyruvate kinase-2 (*Homo sapiens*) (Spot 2), and glyceraldehyde-3-phosphate dehydrogenase isoform 2 (*Homo sapiens*) (Spot 3).

*Spot 1*

 1 MAVMRTLRAM AMQKIFAREI LDSRGNPTVE VDLHTAKGRF RAAVPSGAST
 51 GIYEALELRD GDKGRYLGKG VLKAVENINN TLGPALLQKK LSVVDQEKVD
 101 KFMIELDGTE NKSKFGANAI LGVSLAVCKA GAAEKGVPLY RHIADLAGNP
 151 DLILPVPAFN VINGGSHAGN KLAMQEFMIL PVGASSFKEA MRIGAEVYHH
 201 LKGVIKAKYG KDATNVGDEG GFAPNILENN EALELLKTAI QAAGYPDKVV
 251 IGMDVAASEF YRNGKTDLF KSPDDPARHI TGEKLGELYK SFIKNYPGEA
 301 FGCPSVPARI PCSCLIY.

*Spot 2*

 1 MSKPHSEAGT AFIQTQQLHA AMADTFLEHM CRLDIDSPPI TARNTGIICT
 51 IGPASRSVET LKEMIKSGMN VARLNFSHGT HEYHAETIKN VRTATESFAS
 101 DPILYRPVAV ALDTKGPEIR TGLIKGSGTA EVELKKGATL KITLDNAYME
 151 KCDENILWLD YKNICKVVEV GSKIYVDDGL ISLQVKQKGA DFLVTEVENG
 201 GSLGSKKGVN LPGAAVDLPA VSEKDIQDLK FGVEQDVDMV FASFIRKASD
 251 VHEVRKVLGE KGKNIKIISK IENHEGVRRF DEILEASDGI MVARGDLGIE
 301 IPAEKVFLAQ KMMIGRCNRA GKPVICATQM LESMIKKPPP TRAEGSDVAN
 351 AVLDGADCIM LSGETAKGDY PLEAVRMQLH IAREAEAAIY HLQLFEELRR
 401 LAPITSDPTE ATAVGAVEAS FKCCSGAIIV LTKSGRSAHQ VARYRPRAPI
 451 IAVTRNPQTA RQAHLYRGIF PVLCKDPVQE AWAEDVDLRV NFAMNVGKAR
 501 GFFKKGDVVI VLTGWRPGSG FTNTMRVVPV P.

*Spot 3*

 1 MGKVKVGVNG FGRIGRLVTR AAFNSGKVDI VAINDPFIDL NYMVYMFQYD
 51 STHGKFHGTV KAENGKLVIN GNPITIFQER DPSKIKWGDA GAEYVVESTG
 101 VFTTMEKAGA HLQGGAKRVI ISAPSADAPM FVMGVNHEKY DNSLKIISNA
 151 SCTTNCLAPL AKVIHDNFGI VEGLMTTVHA ITATQKTVDG PSGKLWRDGR
 201 GALQNIIPAS TGAAKAVGKV IPELDGKLTG MAFRVPTANV SVVDLTCRLE
 251 KPAKYDDIKK VVKQASEGPL KGILGYTEHQ VVSSDFNSDT HSSTFDAGAG
 301 IALNDHFVKL ISWYDNEFGY SNRVVDLMAH MASKE.

## 4. Discussion and Conclusion

It is well documented that cancer is a critical health problem and one of the major causes of death all over the world. The high mortality rate is associated with the limited availability of proper screening and early detection methods, as well as poor access to treatment, especially in Africa (Global Cancer: Facts and Figures, 2011) [15]. Drug discovery from medicinal plants continues to provide new and important leads against cancer, which makes the isolation and characterization of pharmacologically active compounds from medicinal plants a continuous process [16]. Due to the considerable interest in the chemotherapeutic treatment of cancer, a great deal of effort has been put in the identification of highly effective and less toxic anticancer drugs.

Preliminary and unpublished studies in our laboratory have shown the acetone leaf extract of DL to possess antioxidant and antiproliferative properties against human leukaemia (HL-60) cell lines. This finding has prompted us to further investigate the effect on this extract on MCF-7 cells. In this study, the acetone leaf extract of DL was qualitatively and quantitatively evaluated for its antioxidant activity. In the qualitative assay, constituent compounds were separated on TLC plates using solvent systems of different polarities and sprayed with DPPH. Compounds with antioxidant activity react with DPPH with a resultant formation of a yellow band against a purple background. Eluted plates sprayed with DPPH showed the presence of constituents in the extract with high antioxidant activity as evidenced by the high intensity of the yellow bands obtained. Quantitative assessment of the antioxidant constituents using the DPPH scavenging assay showed the extract to inhibit the generation of free radicals in a concentration-dependent manner with concentrations of 250 *μ*g/mL and above exhibiting more than 50% free radical scavenging activity.

Nitric oxide is a highly diffusible, lipophilic, and physiological messenger that is responsible for the regulation of numerous important physiological responses including respiration, dilation of blood vessels, immune response, cell migration, and apoptosis. Its various mode of action in the tumour environment is related to heterogeneous cell responses with particular attention in the regulation of the stress response mediated by the hypoxia inducible factor-1 and* p*53, generally leading to growth arrest, apoptosis, or adaptation [17]. As such, the ability of the extract to decrease the production of nitric oxide was also assessed. The assay is based on the principle that sodium nitroprusside, in aqueous solution, generates nitric oxide which then interacts with oxygen to produce nitrite ions (free radicals) that can be measured by Griess reagent. The extract at high concentration was shown to decrease the amount of nitric oxide production within the test system. Scavengers of nitric oxide compete with oxygen leading to a reduced production of nitrite ions [18, 19]. Indeed, studies abound that suggest the role of relatively low concentrations of nitric oxide in favouring cell proliferation and antiapoptotic responses while higher levels are considered to favour pathways that induce cell-cycle arrest, mitochondria respiration, senescence, or apoptosis [20].

In light of this, the extract was then evaluated for its antiproliferative effect on the growth of MCF-7 (breast) cancer cell lines. The extract was shown to decrease the viability of these cells in a concentration- and time-dependent manner. The IC_50_ value corresponding to the log concentration of 1.8, equivalent to 63 *μ*g/mL, was determined. Although the cells were exposed to various concentrations of the extract for a fixed period of time, cells exposed for 24 h showed a high percentage of survival at the various concentrations of the extract used, with decrease in viability at extended time of exposure, a finding that is suggestive that extended time of incubation results in a build-up of nitric oxide production within the test system. Nitric oxide is produced by inducible nitric oxide synthase (iNOS) and plays an important role in the cytotoxicity of cancer cells. Its mediated cytotoxicity has also been shown to inhibit mitochondrial respiration and DNA synthesis in cell targets, including breast cancer cells [21]. Nitric oxide also serves in a negative feedback loop in antagonizing the prolong activation of NF-*κ*B, thereby limiting cancer cell survival [22]. Other authors, in an effort to assess the amount of nitric oxide produced in MCF-7 cells treated with a glycoprotein isolated from* Solanum nigrum* L. observed an increase in endogenous nitric oxide production in a dose-dependent manner [23]. Curcumin, used as the positive control, is a major component of the spice turmeric and has been reported to possess antitumour, anti-inflammatory, and antioxidant properties [24, 25], including the inhibition of the proliferation of several tumour cells [24, 26]. The DL extract at the highest concentration (500 *μ*g/mL) was shown to be more potent than curcumin (7.36 *μ*g/mL), suggesting the cytotoxicity of the extract and curcumin to be concentration and time of exposure-dependent.

Currently, there are several techniques and markers used in research for the detection of apoptosis* in vitro* and these include the frequently applied* in vitro* detection method using annexin V and DNA-binding dye, Hoechst 335258. Cells undergoing apoptosis have the propensity of losing their membrane asymmetry in the early stages of apoptosis, chromatin condensation, and forming apoptotic bodies [27]. In order to characterise the mode of cell death associated with DL growth inhibitory effects on the MCF-7 cells, the morphological changes associated with apoptosis were assessed. Indeed, the MCF-7 cells demonstrated chromatin condensation and plasma membrane blebbing following exposure to the extract for 48 h. The chromatin of the untreated cells remained intact as indicated by the even spread of the Hoechst dye, suggesting that DL growth inhibitory activity was due to the induction of apoptosis.

Furthermore, to quantitatively ascertain the percentage of cells within the population that were actively undergoing apoptosis, untreated and treated MCF-7 cells were stained using annexin V-FITC/propidium iodide (PI) and evaluated by flow cytometry. The results revealed that about 50% of curcumin-treated cells were annexin V-positive/PI-negative following 24 h of exposure. On the other hand, approximately 70% of the extract-treated cells were annexin V-positive/PI-negative following 48 h of exposure. However, about 20% of the cells were shown to stain annexin V-positive/PI-positive as a result of late apoptosis. Annexin V-negative/PI-positive cells were also shown to emerge after 48 h of exposure to extract, possibly due to loss of necrotic plasma membrane during the staining procedure. These results demonstrate that not only apoptotic but also primary necrotic cells show annexin V-positive/PI-negative staining before they become PI-positive. These findings buttress the notion that the growth inhibitory effects of the DL extract is associated with the induction of apoptosis in MCF-7 breast cancer cells. The induction or activation of apoptosis can occur if cells are exposed or treated with a cytotoxic drug or when cells encounter a specific death-inducing signal [28].

In order to further investigate the mechanism(s) of action involved in the induction of apoptosis by the DL extract, the mRNA expression levels of apoptosis-related genes were assessed by quantitative real-time PCR. The targeted apoptotic-related genes in this study were the* p*53,* bax, bcl*-2, and* p*21. As much as the first three of these genes gave plausible data in this study, a good amplification of the* p*21 gene was, however, not achieved in this study and as such, the results for this gene are omitted in this report.

Expression of* p*21 has been shown to be upregulated by the* p*53 tumour suppressor gene* in vitro* in response to DNA-damaging agents and is indeed considered as a function that may be critical for its tumour suppressor properties. Hence the putative role of* p*21 in growth arrest mediated by* p*53 makes it an important gene with respect to the understanding of loss of growth control in transformation processes. Other authors [29] have shown that* p*21 can be regulated independently of* p*53 in several situations including during normal tissue development, following serum stimulation, and during cellular differentiation in mice. Thus, the possibility that either* p*21 may not be completely dependent on* p*53 in certain cells* in vitro* for apoptosis to occur or certain substances present in the extract influenced its nonexpression may not be ruled out. Further studies are however required to buttress this assumption.

The current findings revealed that the DL extract, at various concentrations, increased the fold change or upregulated the expression of* p*53, beyond 24 h of exposure in a concentration-dependent manner. The role of the tumour suppressor* p*53 in regulating the expression levels of genes that mediate cell division cycle arrest and/or classical apoptosis in response to cellular stresses, including DNA damage, growth factor deprivation, hypoxia, and oncogene activation, has been well documented [30].

*p*53, as a transcription factor, is also considered to regulate the expression levels of pro- and antiapoptotic family members [31]. The downregulation of* bcl*-2 expression by the DL extract was shown to be in a concentration- and time-dependent manner. Bcl-2 is an antiapoptotic protein that is localised in the mitochondrial membrane and promotes cell survival causing the cells to accumulate in the G1/G0 interphase, thus protecting them from cell death [32]. As such, the downregulation of* Bcl*-2 leads to minimal inhibition of the apoptotic pathway. In contrast, the proapoptotic protein Bax, is antagonised by the antiapoptotic Bcl-2 protein. In response to an apoptotic stimulus, it has been found that Bax becomes activated and translocates to the mitochondria where it prevents the antiapoptotic Bcl*-*2 from inhibiting the proapoptotic proteins ([33, 34]; a report that supports the finding in this study that shows the upregulation of Bax.

Following the assessment of the gene expression levels using qrt-PCR, the differentially expressed proteins under the treatment conditions were separated and identified by 2D electrophoresis (2DE) and mass spectrometry (MS). The idea of using 2DE was to separate proteins in a sample using two independent properties such as isoelectric point and mass [35]. The identified spots represent protein size and amount present in the cells. The spots on gels were analysed by MALDI-TOF mass spectrometry and subjected to tryptic digestion and subsequent peptide sequencing. The obtained sequences matched three proteins/enzymes that are mostly utilised in carbohydrate metabolism, namely, enolase, pyruvate kinase (PK), and glyceraldehyde-3-phosphate (GAPDH).

The detected proteins are predominantly involved in certain biological pathways such as glycolysis and carbon metabolism. Pyruvate kinase is an enzyme that catalyses the transfer of a phosphoryl group from phosphoenolpyruvate to adenosine diphosphate (ADP) generating adenosine triphosphate (ATP) and pyruvate [36, 37]. Although the enzyme pyruvate kinase is mainly involved in glycolysis, its activity has been reported to increase in malignant tumours including those of breast tissues [38]. Clinical studies show that the presence of pyruvate kinase in breast cancer patients is essential. This enzyme is used as a marker in trastuzumab dual therapy treatment of breast cancer [39]. Trastuzumab, commonly known as Herceptin, is a monoclonal antibody used in the treatment of certain breast cancers. Therefore, the expression of this enzyme in MCF-7 cell lines is key in the diagnosis and treatment monitoring of breast tumours. In this study pyruvate kinase was shown to be highly expressed in the cells.

Enolase on the other hand is a gene that encodes one of the three enolase isoenzymes found in mammals. Its function is to catalyse dehydration of 2-phospho-D-glycerate to phosphoenolpyruvate in the second phase of the glycolytic pathway. The expression of the three isoforms with high sequence identity is tissue specific: *α*-enolase is present in adult tissue,*β*-enolase in muscle tissue, and gamma *γ*-enolase in neurons and neuroendocrine tissues. The *β*-isoform, found in skeletal muscle, plays a key role in muscle development and regeneration [40]. Although the identified protein in this study was the *β*-isoform of enolase, the *α*-enolase is found to be expressed on the cell surface in tumours where it acts as a receptor, promoting cell migration and cancer metastasis. Its expression is increased or upregulated at the mRNA and protein levels in several tumours including the cervix, colon, and breast [41]. This is supported by the intensity of its counterpart protein, *β*-enolase, identified in this study. The increased expression of *α*-enolase protein is considered to influence chemotherapeutic treatments in estrogen receptor-positive breast tumours where it induces drug resistance [42].

On the other hand, glyceraldehyde-3-phosphate (GAPDH) is a constitutive or housekeeping gene required for the maintenance of basic cellular function and is expressed in all cells of organisms under normal physiological conditions. It plays a notable role in gene transcription and DNA repair [43]. The constant expression levels of GAPDH proteins are thus required in all cells in order to maintain normal metabolic functions. The variations in the expression levels of the detected protein observed in this study could be as a result of cell proliferation, arrest, and altered cell morphology.

In conclusion, findings in this study show the acetone leaf extract of DL to possess antioxidant activity both as a reducing agent and as a free radical scavenger. Our data also revealed that the DL extract exerts its antiproliferative effects by inducing apoptosis in MCF-7 breast cancer cells in a* p*53-dependent manner. The mutation of* p*53 is considered to be one of the contributing factors in the development of various forms of cancer. Hence, the ability of the extract to exert its antiproliferative effect on MCF-7 cancer cells in a* p*53-dependent pathway makes DL a potential source for identification of lead or novel compounds that can be explored for the prevention, treatment, and management of different types of cancer. The extract was also shown to induce the upregulation of* Bax* and downregulate* Bcl*-2 mRNA expression levels, key genes in programmed cell death events. Further studies are ongoing to investigate whether the effect of the extract on* p*21 expression is* p*53-dependent in MCF-7 cells as well as its effect on caspase activity and other apoptotic proteins using proteomics and the isolation of the active constituent(s) responsible for the observed activities.

## Figures and Tables

**Table 1:** Primer sequences used in real-time PCR [9].

*bax*	Forward primer:	5′-GGGTGGTTGGGTGAGACTC-3′
	Reverse primer:	5′-AGACACGTAAGGAAAACGCATTA-3′

*bcl-2*	Forward primer:	5′-CTGTCTGGAATAGAAGGCACTCT-3′
	Reverse primer:	5′-ACCCTCGTCTTTTAGAAACAGGA-3′

*p21*	Forward primer:	5′-TGCAACATTTTCGGCAGCTAA-3′
	Reverse primer:	5′-TCCTCAAATTCGTCAAAGGGTTC-3′

*p53*	Forward primer:	5′-ATTGCCAGAGGTTTTACCGAG-3′
	Reverse primer:	5′-CGAAACTCCCACGGATAGAATCT-3′

*GAPDH*	Forward primer:	5′-ACCCACTCCTCCACCTTTG-3′
	Reverse primer:	5′-CTCTTGTGCTCTTGCTGGG-3′

**Table 2:** A summary of the identification results from the spots excised from coomassie-stained 2D gels from proteins isolated from MCF-7 cells.

Spot	BLASTed Pep. seq	NCBI accession	MW (kDa)	pI	Score	Identified protein
1	AAVPSGASTGIYEALELR	EAW90376	34	8.19	56.6 bits (126)	Enolase 3

2	IENHEGVRRFDEILEASDGIMVAR	AAH94767	58	7.58	81.2 bits (184)	PKM2

3	LISWYDNEFGYSNR	NP_001243728	34	8.26	54.9 bits (122)	GAPDH

Spot 1: enolase-3 (beta-muscle) isoform CRA_b (*Homo sapiens*).

Spot 2: pyruvate kinase-2, PKM2 (*Homo sapiens*).

Spot 3: glyceraldehyde-3-phosphate dehydrogenase isoform 2, GAPDH (*Homo sapiens*).
